# 

**Published:** 1896-04

**Authors:** 


### CONTENTS FOR APRIL, 1896.

ORIGINAL COMMUNICATIONS. PAGE.

First Uses of Chloroform and Ether in Buffalo. By JOHN HAUENSTEIN, M. D. .......... 689

Chloroform—Its Method of Administration, Its Dangers and Their Treatment. By JOHN PARMENTER, M. D. .......... 695

Medical Surgery. By G. E. BENNINGHOFF, M. D. .......... 710

Vaginal Celiotomy. By WM. B. JONES, M. D. .......... 715

PROGRESS IN MEDICAL SCIENCE.

Diseases of Nose and Throat .......... 722

Medicine, Pathology and Therapeutics .......... 728

Medical Education and State Medical Examinations .......... 734

EDITORIAL.

Jubilee of the Medical Department, University of Buffalo .......... 743

Topics of the Month .......... 745

Personal .......... 749

Obituary .......... 750

Society Meetings .......... 751

Medical College and Hospital Notes .......... 753

Reviews .......... 754

Miscellany .......... 767

Literary Notes .......... 765

